# Role of “Stiff Rim” sign obtained by shear wave elastography in diagnosis and guiding therapy of breast cancer

**DOI:** 10.7150/ijms.64243

**Published:** 2021-08-28

**Authors:** Yan-jun Xu, Hui-ling Gong, Bin Hu, Bing Hu

**Affiliations:** 1 Department of Ultrasound in Medicine, Shanghai Jiao Tong University Affiliated Sixth People's Hospital, Shanghai Institute of Ultrasound in Medicine, Shanghai 200233, P.R, China; 2 Department of Ultrasound, Minhang Hospital, Fudan University, Shanghai 201199, P.R, China

**Keywords:** stiff rim sign, shear wave elastography, breast lesion, BI-RADS, TNM stage

## Abstract

**Background:** Because the halo around the tumor in shear wave elastography (SWE) is defined as the “stiff rim” sign, the diagnosis of breast lesions with the stiff rim sign is popular. However, only a few studies have described the stiff rim sign quantitatively.

**Objective:** This study aimed to investigate the usefulness of the stiff rim sign in the diagnosis and tumor, node, metastasis stage of breast cancer.

**Methods:** Two hundred and ten breast lesions were analyzed retrospectively. The maximum, mean, minimum Young's modulus (YM), and the YM standard deviation in the lesion, the peritumoral stiffness (shell), and the region containing lesion and shell were obtained. The suspicious SWE feature with the best diagnostic performance was chosen to downgrade or upgrade the Breast Imaging Reporting and Data System (BI-RADS) classification. The coincidence rates of SWE and B-mode ultrasound in T staging and their positive predictive value (PPV) for T staging were compared.

**Results:** The presence of “stiff rim” sign was selected to upgrade or downgrade the BI-RADS classification because of its best performance. In pathological benign lesions, 18.9% (25 of 132) of lesions should undergo biopsy if BI-RADS combined with the stiff rim sign were referred while it was 57.6% (76 of 132) if BI-RADS alone was referred. The coincidence rate of T2 staging evaluated by SWE was significantly higher than B-mode ultrasound (about 30% increase, *P* < 0.001). The PPVs of SWE for T1 and T2 staging were higher than B-mode ultrasound (*P* < 0.05).

**Conclusions:** BI-RADS combined with “stiff rim” sign is expected to improve the diagnostic performance of breast lesions to avoid unnecessary biopsy. The maximum diameter of the lesion measured in SWE is more accurate than B-mode ultrasound in the estimation of T staging, which is beneficial to the treatment and prognosis of breast cancer.

## Introduction

Ultrasound elastography has become the most important adjunct to ultrasonography in the diagnosis of breast cancer. It is recognized that adding elastography features to Breast Imaging Reporting and Data System (BI-RADS) classification is valuable for the diagnosis of breast lesions 1. Several studies have confirmed that shear wave elastography (SWE) combined with ultrasonography shows high accuracy in the differential diagnosis of benign and malignant breast diseases 2-4. However, so far none of the diagnostic criteria based on SWE is satisfactory. Malignant breast tumors normally have significantly higher elasticity values than benign breast masses. But some intratumoral areas with conflicted features (such as increased elasticity caused by calcification in benign breast masses or decreased elasticity caused by necrosis in malignant tumors) may lead to misdiagnosis 5-7. Recent research reported that the maximum area of stiffness in malignant tumors was always found in the peritumoral stroma rather than inside the cancer 8, 9. Since Zhou et al. 10 first called peritumoral stiffness the “stiff rim” sign, the research on the diagnosis of breast lesions with the stiff rim sign has become an important research focus. Desmoplastic reaction and tumor cell infiltration into the peritumoral stroma have been proven to be the possible mechanism of the stiff rim sign 11. Hence, it may be more helpful than intratumoral stiffness in the diagnosis of breast lesions.

Nevertheless, to date, the application of the stiff rim sign is still limited. Only a few available studies have described the stiff rim sign of breast masses, which are considered to indicate the risk of malignancy 12, 13. More detailed research is needed to explore the role of the stiff rim sign in the diagnosis and treatment of breast lesions. In this study, by quantitatively analyzing the stiff rim sign around breast masses, we investigated the usefulness of the stiff rim sign in the diagnosis and tumor, node, metastasis (TNM) stage of breast cancer.

## Materials and methods

This retrospective study was conducted following the Declaration of Helsinki and the Declaration of Istanbul. All procedures were approved by the Institutional Review Board at Minhang Hospital, Fudan University. Informed consents were obtained from all patients at the time of their examinations.

### Patients

**Figure 1:**
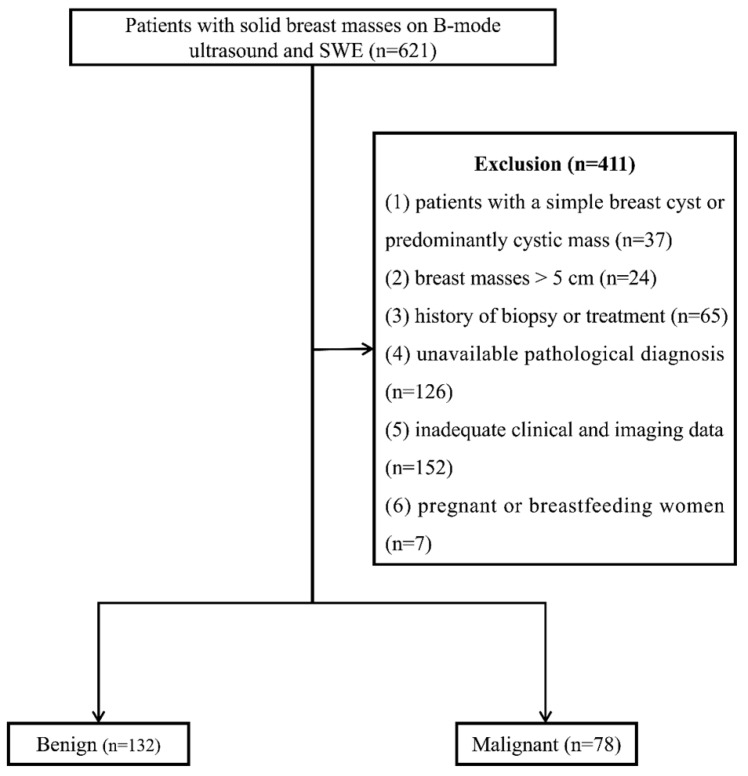
Flow chart of patient enrollment. SWE: shear wave elastography.

From January 2016 to January 2021, 621 consecutive female patients with solid breast masses were examined with B-mode ultrasound and SWE, which was the routine breast ultrasound examination protocol at our institution. Four hundred and eleven patients were excluded for the following reasons: (1) patients with a simple breast cyst or predominantly cystic mass (n=37), (2) breast masses > 5 cm (n=24), (3) history of biopsy or treatment (n=65), (4) unavailable pathological diagnosis (n=126), (5) inadequate clinical and imaging data (n=152), (6) pregnant or breastfeeding women (n=7). One lesion per patient was included, and the largest lesion was chosen for multiple lesions. Finally, 210 breast lesions in 210 women (47.29 ± 12.91 years) were enrolled (**Figure 1**).

### Image acquisition

Gray-scale and SWE images were obtained during a standard ultrasound examination using a Resona 7 ultrasound system (Mindray Medical International, Shenzhen, P.R.China), which was performed by one of two sonographers with more than 10 years of experience in breast ultrasonography. At least two orthogonal grayscale images were obtained for each solid breast mass, with the patient in the supine position. These images were classified by BI-RADS according to the shape, margin, boundary, echogenicity, posterior acoustic feature, color Doppler flow signal, presence of calcification, and other characteristics of the tumor 14.

**Figure 2:**
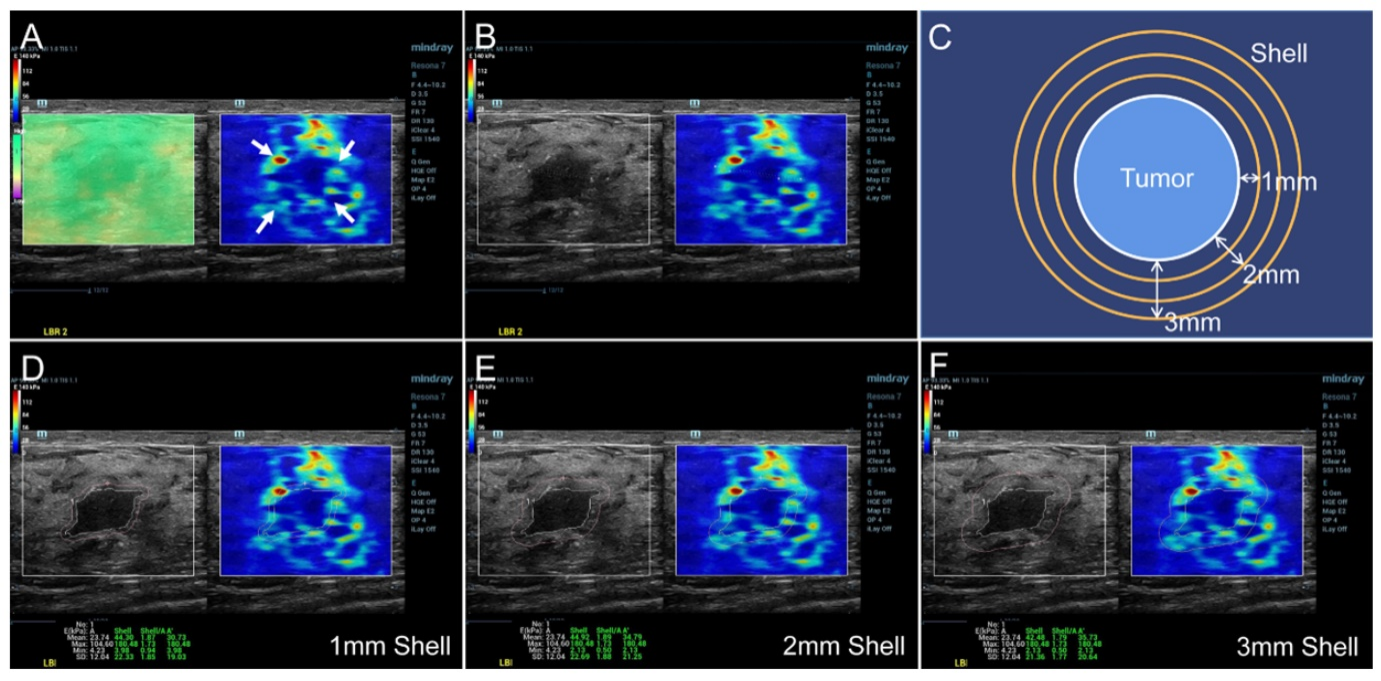
SWE dual dynamic mode of invasive ductal carcinoma. A satisfied SWE image quality was obtained before quantitative analyses (**Figure 2A** left, represented as uniform green). The red and orange halo around the tumor suggested the stiff rim sign (Figure 2A right, marked as arrows). The maximum diameter of lesion was then measured in the SWE dual dynamic mode (**Figure 2B**). A sketch map of the stiff rim sign was presented in **Figure 2C**. The ultrasound system would automatically plot the shells around the tumor with a width of 1-3 mm. When the tumor boundary was delineated manually on the gray-scale map, the system calculated the YM values of the lesion, 1-3 mm shell, and the area contained the lesion and the shell (**Figure 2D-F**). SWE: shear wave elastography, YM: Young's modulus.

The SWE mode was used after the standard ultrasound examination. The patients were required to hold their breath for a few seconds and the transducer was put on the surface of breast vertically as gently as possible to reduce artificial stiffness. A rectangular region of interest (ROI) was set to include the whole lesion and adjacent breast tissue in the dual dynamic mode of SWE. The SWE quality mode was run first to ensure high accuracy and repeatability of the measured elasticity. The average displacement of the tissue within the ROI were calculated automatically based on a block matching algorithm in the system, and displayed with different colors. Uniform green without purple artifacts indicated small tissue displacement and high-quality SWE image. The stiffness of the breast lesion was represented by a color map from red to blue (hard to soft) under a SWE display scale ranging between 0 and 140 kPa of YM value (**Figure 2A**). First, the presence of the stiff rim sign around the lesion (red or orange halo) was observed (**Figure 2A**). The maximum diameter of the lesion was then measured in the dual dynamic mode of SWE after the largest section of the lesion was determined (**Figure 2B**). After that, the tumor contour was delineated manually. Three layers (1-3 mm) of peritumoral stiffness (shell) around the lesion were plotted by the system (**Figure 2C**). The maximum, mean, minimum Young's modulus (YM), and the YM standard deviation in lesion, shell, and the region containing lesion and shell were calculated automatically. They were marked as E_max_, E_mean_, E_min_, and E_sd_ for lesion; Es_max_, Es_mean_, Es_min_, and Es_sd_ for shell; and Els_max_, Els_mean,_ Els_min_, and Els_sd_ for lesion plus shell, respectively (**Figure 2D-F**). The Es and Els of different shell thicknesses were distinguished by *n* (*n* = 1, 2, 3 mm), namely Es*_n_* and Els*_n_* (eg, Es_1,max_ and Els_1,max_ for 1 mm shell).

### Histopathologic examination

A histopathologic diagnosis, which was regarded as the standard reference, was obtained from surgical excision, and otherwise from core biopsy by a pathologist with more than 10 years of experience who was blinded to the ultrasound results. The pathologic features, including tumor type, size (measured from gross pathology), histologic grade, vascular invasion status, and lymph node status, were recorded. The tumors were classified according to the 7th edition of breast TNM staging 15.

### Statistical analysis

The BI-RADS, stiff rim sign, and the YM values of each position of the lesion between benign and malignant tumors were compared using the Chi-squared test and Mann-Whitney U test. The diagnostic accuracy, sensitivity, and specificity of each variable were evaluated by the receiver operator characteristic (ROC) curve. The suspicious SWE feature with the highest area under the curve (AUC) was chosen to downgrade or upgrade the BI-RADS classification (to downgrade if the SWE feature was absent or upgrade if it was present). BI-RADS 3 would not be downgraded while BI-RADS 5 would not be upgraded. The correlations between the tumor size and the size measured on gray-scale and SWE images were evaluated by Pearson's correlation analysis. The coincidence rates of SWE and B-mode ultrasound in pathological T (pT) stage and their positive predictive value (PPV) for pT stage were compared (T staging for breast cancer: T1 ≤ 20mm; T2: >20 mm & ≤50 mm; T3: >50 mm). Statistical analyses were performed using IBM SPSS Statistics 22.0 (IBM Corp., Armonk, NY, United States), and Medcalc (Version 22.0.1; MedCalc Software, Ostend, Belgium).

## Results

### Pathological findings

Two hundred and ten breast lesions were collected in this study, including 132 benign lesions and 78 malignant ones. The maximum diameter of the malignant lesions [27.2 (17.5, 35.5) mm] was greater than the benign ones [17.5 (12.0, 23.5) mm] (*P* < 0.001). The summary of pathological diagnosis of benign and malignant tumors under different BI-RADS classification was described in **Table 1**.

### Diagnostic performance of SWE features

**Figure 3:**
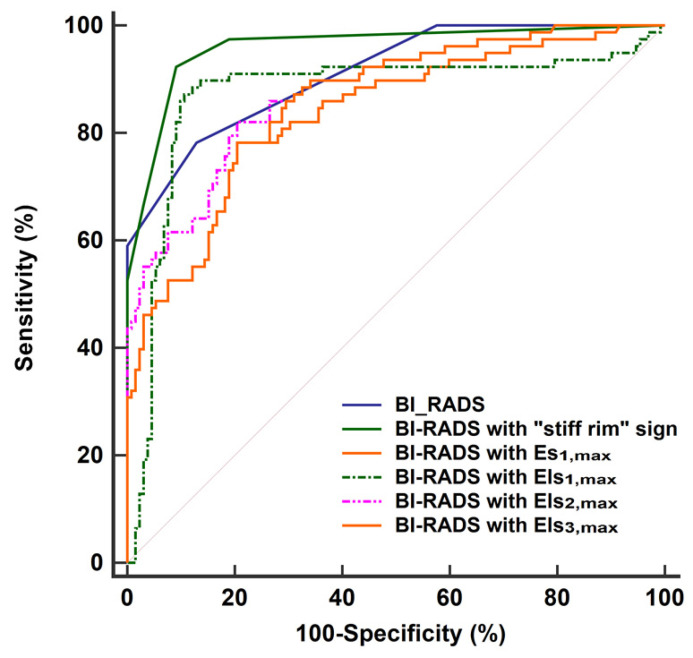
ROC curves of BI-RADS and the combinations of BI-RADS and SWE features in the diagnosis of breast lesions. The AUC of BI-RADS was 0.911 (95% CI: 0.864 to 0.946). It increased to 0.946 (95% CI: 0.924 to 0.982) when BI-RADS combined with “stiff rim” sign, which was significantly higher than other combinations including BI-RADS with Es_1,max_ (AUC: 0.835, 95% CI: 0.777 to 0.882), Els_1,max_ (AUC: 0.872, 95% CI: 0.819 to 0.914), Els_2,max_ (AUC: 0.876, 95% CI: 0.824 to 0.918), and Els_3_,_max_ (AUC: 0.855, 95% CI: 0.800 to 0.899). ROC: receiver operator characteristic, BI-RADS: breast imaging reporting and data system. AUC: area under the curve, CI: confidence interval. The maximum Young's modulus in lesion, shell, and the region containing lesion and shell are marked as E_max_ for lesion, Es_max_ for shell, and Els_max_ for lesion plus shell, respectively. The Es and Els of different thickness of shell are represented by *n* (*n* = 1,2,3), namely Es*_n_* and Els*_n_* (eg, Es_1,max_ and Els_1,max_ for 1mm shell).

The diagnostic performances of the quantitative features concerning SWE were shown in **Table 2**. Except for E_min_, Es_1,min_, Els_1,min_, Es_2,min_, Els_2,min_, Es3_,min_, and Els_3,min_, other SWE features in malignant lesions were higher than in benign lesions (*P* < 0.001 for all). According to the ROC analyses, the stiff rim sign, Es_1,max_, Els_1,max_, Els_2,max_, and Els_3,max_ showed higher diagnostic performances than other SWE parameters (*AUC* > 0.8 for all). Among them, the stiff rim sign was selected to upgrade or downgrade the BI-RADS classification since it had the highest *AUC* (0.849). **Figure 3** shows that BI-RADS combined with the stiff rim sign could increase the diagnostic accuracy to 0.946, which was significantly higher than other combinations including BI-RADS with Es_1,max_ (AUC: 0.835), Els_1,max_ (AUC: 0.872), Els_2,max_ (AUC: 0.876), and Els_3,max_ (AUC: 0.855) (*P* < 0.001 for all).

### Hypothetical effect of BI-RADS in combination with the stiff rim sign

As shown in **Table 3**, among the 76 BI-RADS 4a lesions based on B-mode ultrasound, 51 were downgraded to BI-RADS 3, and 25 were upgraded to 4b. For the 32 BI-RADS 4b lesions, 23 were downgraded to 4a, and 9 were upgraded to 4c. For the 31 BI-RADS 4c lesions, 5 were downgraded to 4b, and 26 were upgraded to 4c. Among pathological benign lesions, 57.6% (76 of 132) of lesions should undergo needle biopsy according to the ultrasound-based BI-RADS. The corresponding percentage was 18.9% (25 of 132) according to the combination. In the assessment of malignant lesions, 100.0% of lesions (78 of 78) could be correctly screened for needle biopsy by both the ultrasound-based BI-RADS and the combination.

### Correlation between the tumor size and the size measured on gray-scale and SWE images

**Figure 4:**
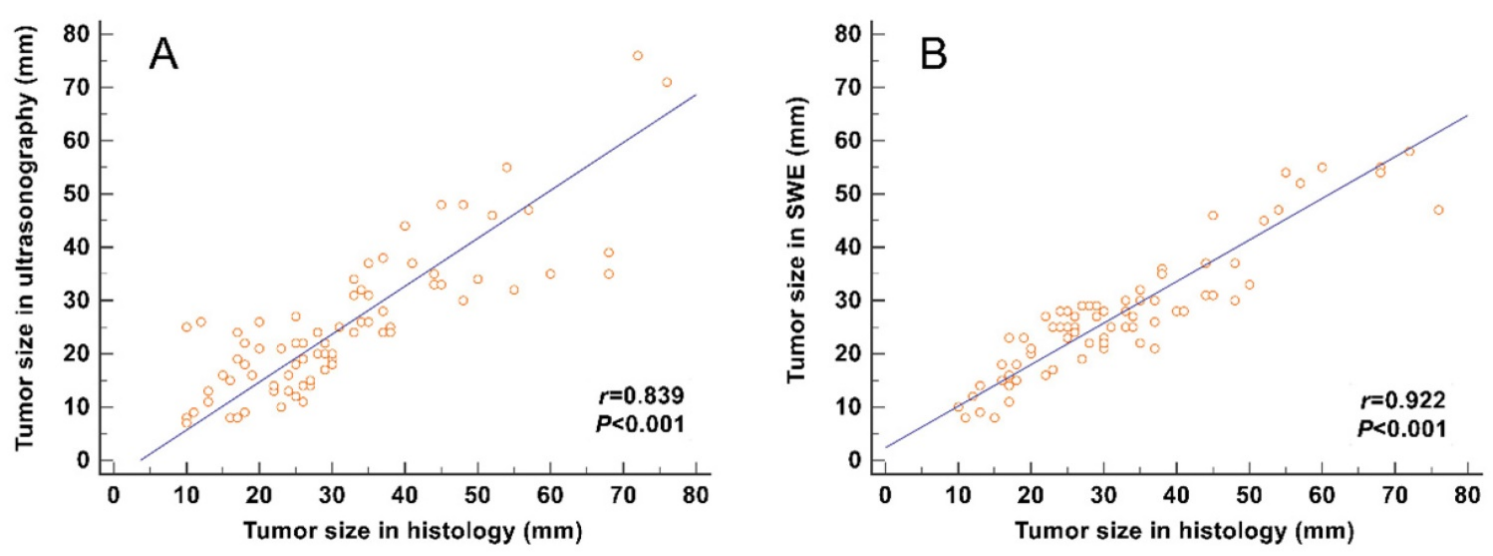
Correlation analyses between the tumor size measured from gross pathology and the size measured on gray-scale and SWE images. The correlation of the size determined in SWE images (including the stiff rim sign) (**A**) was higher than that in gray-scale ultrasound images (**B**). SWE: shear wave elastography.

The correlation between the tumor size and the maximum size measured on SWE images (including the stiff rim sign) (*r* = 0.922) was higher than that on gray-scale ultrasound images (*r* = 0.839) (**Figure 4**).

### Comparison of SWE and ultrasound in evaluating T staging of breast cancer

The total coincidence rate of T staging evaluated by SWE (87.2%) was higher than that by conventional ultrasound (58.9%, *P* < 0.001). Furthermore, the coincidence rate of T2 staging evaluated by SWE was significantly higher than ultrasound (about 30% increase, *P* < 0.001) (**Table 4**). The PPVs of SWE for T1 and T2 staging were higher than ultrasound (*P* < 0.05), while they were similar in the T3 staging (**Table 5**).

## Discussion

In the present study, the “stiff rim” sign and several SWE quantitative features (Es_1,max_, Els_1,max_, Els_2,max_, and Els_3,max_) were shown to be useful in diagnosing breast lesions, among which the stiff rim sign presented the highest accuracy. The diagnostic accuracy would be further improved when BI-RADS combined with the stiff rim sign, although the accuracy based on the stiff rim sign alone was not superior to BI-RADS. Furthermore, the coincidence rate of the maximum tumor size measured on SWE images and the tumor size measured from gross pathology was higher compared with that in B-mode ultrasound images (breast cancer with the stiff rim sign tend to be larger), which made the T staging estimation in SWE images more accurate. It is beneficial to the preoperative assessment of breast cancer and the decision in therapy selection.

Although SWE features are regarded to improve the differential diagnosis of breast lesions, it is still unclear which feature has the best diagnostic performance 16. The research of Berg et al. 4, Evans et al. 9, and Wang et al. 17 reported that the E_max_ and E_mean_ within the tumor were most helpful for breast-lesion characterization. Zhou et al. 10, Shi et al. 18, and Çebi et al. 19 found that E_sd_ and elasticity ratio showed similar performance with E_max_. This inconsistency may be partly due to the different locations where the stiffness is measured.

It is known that the area with the maximum stiffness is often in the shell of the tumor 20. The desmoplastic reaction caused by the peritumoral infiltration of cancer cells into the interstitial tissues increases the peritumoral stiffness 9, 21, 22. Recent studies using this shell-based analysis found that the E_max_ of 1-3mm shell was the most accurate for differential diagnosis of benign and malignant breast lesions 20, 23-25. Our study offered the automatically plotted 1-3 mm shells around the breast lesion, which helped to calculate the maximum YM at the tumor border. However, unlike previous studies 20, 23-25, our results revealed that the presence of the stiff rim sign had the highest diagnostic accuracy rather than those quantitative SWE parameters, especially the E_max_ of 1-3mm shell. We supposed that this is because the shell, which is composed of glandular tissue, may also exist around benign tumors, thus affecting the diagnostic performance of the E_max_ at the shell 12. Besides, higher stiffness targets are more difficult to measure accurately because the shear wavelength is longer and the time delays related to the shear wave propagation are harder to estimate reliably.

In the benign lesions, 38.6% (51 of 132) lesions defined as BI-RADS 4a could be downgraded to BI-RADS 3 to avoid unnecessary puncture biopsy if combined with the stiff rim sign. In the malignant lesions regardless of what method was adopted, all lesions could be correctly selected for biopsy. However, 3 benign lesions (3 fibroadenomas) were incorrectly upgraded to BI-RADS 4b from 4a. There may be two explanations for this wrong upgrade. The first explanation is that sometimes an incomplete halo around the lesion is also considered as the stiff rim sign. The second explanation is that the halo artifacts may appear around larger fibroadenomas, where the sclerosed and hyperplastic tissue will increase the shear wave velocity.

In addition to the application of “stiff rim” sign in the diagnosis of breast cancer, its role in the TNM staging has been rarely mentioned. The TNM staging system for breast cancer is an important basis for individualized treatment regimens and prognostic assessment. Accurate preoperative evaluation of T staging is the key to complete tumor resection 26. With the development from radical mastectomy to breast-conserving surgery, achieving the best clinical outcome with minimal treatment has become the goal of the surgical treatment of breast cancer. It is important to accurately determine the tumor size before surgery 27. Our study found that the tumor size measured on gray-scale or SWE images was positively correlated with the postoperative size, which was consistent with the study of Fornage et al. 28. Since the malignant lesions with a stiff rim sign tended to be larger, the tumor size on the SWE image was larger than on the gray-scale image, and the correlation of SWE was higher. This may be related to the invasion of tumor cells into the surrounding tissue and abnormal collagen formation. It is difficult to observe the obvious changes on the gray-scale image. However, it can be displayed on the SWE image because the change in elastic modulus between tissues is much more obvious than the change in acoustic impedance 11, 29. Clinically, "potentially curable breast cancer" refers to tumors that are confined to a local area and can be radically removed by surgery, generally including stage 0, I, and II breast cancer 30. This study found that the total coincidence rate of SWE evaluated T staging and pT staging was higher than that evaluated by B-mode ultrasound, and it was significantly higher in T2 staging. The PPV of SWE in T1 staging was significantly higher than that of ultrasound, although no significant difference in the coincidence rate was found between them. The number of patients in the T3 and T4 staging was too small to allow a definitive conclusion on the coincidence rate and PPV. It indicated that the tumor size measurement on the SWE image (with a stiff rim sign) can more accurately assess the T staging, thereby providing help in decision-making for treatment.

A number of limitations were inevitable in this study. First, inter-observer variability was not taken into account since this was a retrospective study. Second, the determination of the stiff rim sign is subjective to some extent. It may be inaccurate sometimes since some incomplete halo around the lesion would also be recognized. Third, sampling bias may exist due to the single-center study, and large population studies are needed to validate our results.

In conclusion, BI-RADS combined with “stiff rim” sign is expected to improve the diagnostic performance of breast lesions to avoid unnecessary puncture biopsy. The tumor size measured in SWE is more accurate in estimating the T stage than in gray-scale ultrasound since malignant breast tumors with a stiff rim are larger in SWE, which is essential to the prognostic evaluation and optimal treatment in the clinical setting.

## Figures and Tables

**Table 1:** Pathological diagnosis in 210 breast lesions under different BI-RADS classification

	BI-RADS 3 (n=56)		BI-RADS 4a (n=76)		BI-RADS 4b (n=32)		BI-RADS 4c (n=31)		BI-RADS 5 (n=15)	
	Benign (n=56)	Malignant (n=0)	Benign (n=59)	Malignant (n=17)	Benign (n=17)	Malignant (n=15)	Benign (n=0)	Malignant (n=31)	Benign (n=0)	Malignant (n=15)
Pathological diagnosis	Fibroadenoma (n=31)	NA	Fibroadenoma (n=35)	IDC (n=15)	Fibroadenoma (n=12)	IDC (n=13)	NA	IDC (n=24)	NA	IDC (n=13)
	ANDI (n=18)		ANDI (n=16)	DCIS (n=1)	ANDI (n=5)	DCIS (n=2)		DCIS (n=5)		DCIS (n=2)
	Intraductal papilloma (n=7)		Intraductal papilloma (n=6)	ILC (n=1)				ILC (n=1)		
			Chronic inflammation (n=2)					IPC (n=1)		

ANDI: aberrations of normal development and involution, IDC: invasive ductal carcinoma, DCIS: ductal carcinoma in situ, IPC: intraductal papillary carcinoma, ILC: invasive lobular carcinoma

**Table 2:** Diagnostic performance of BI-RADS classification and SWE features

Variables		Benign (n=132)	Malignant (n=78)	*P* value	AUC	Cut-off point	Sensitivity (%)	Specificity (%)
BI-RADS (3-4a / 4b-5)		115/17	17/61	<0.001	0.911	>4a	87.12	78.21
Stiff rim sign (yes / no)		24/118	64/14	<0.001	0.849	Yes	82.05	81.82
E_max_ (kPa)		75.5 (38.9, 95.9)	135.5 (86.1, 187.5)	<0.001	0.751	123	65.8	70.7
E_mean_ (kPa)		22.3 (15.3, 28.2)	27.1 (21.8, 34.2)	0.029	0.647	25.6	62.8	64.4
E_min_ (kPa)		5.3 (3.5, 7.8)	6.2 (4.3, 8.6)	0.071	0.575	5.6	62.8	55.3
E_sd_ (kPa)		11.9 (6.1, 18.2)	17.8 (10.9, 26.2)	0.023	0.688	24.3	32.1	97.7
Shell 1mm	Es_1,max_ (kPa)	79.5 (58.0, 98.6)	138.8 (79.8, 174.5)	<0.001	0.804	107.9	64.1	88.6
	Es_1,mean_ (kPa)	23.7 (13.1, 31.5)	39.1 (33.8, 52.9)	0.014	0.682	38.3	62.9	74.2
	Es_1,min_ (kPa)	4.6 (2.7, 7.1)	5.1 (2.7, 7.6)	0.504	0.528	4.6	57.7	52.3
	Es_1,sd_ (kPa)	14.0 (8.9, 16.8)	23.9 (13.6, 29.5)	0.017	0.731	22.6	51.3	96.2
	Els_1,max_ (kPa)	78.3 (58.0, 98.6)	136.5 (86.1, 187.5)	<0.001	0.815	113.9	66.6	89.1
	Els_1,mean_ (kPa)	22.8 (14.2, 29.5)	32.5 (26.3, 44.7)	0.010	0.695	39.5	65.3	74.4
	Els_1,min_ (kPa)	5.2 (3.2, 7.4)	6.2 (4.3, 8.6)	0.068	0.582	5.4	62.2	54.7
	Els_1,sd_ (kPa)	15.3 (9.2, 17.5)	24.6 (14.5, 30.4)	0.015	0.762	23.5	55.2	94.8
Shell 2mm	Es_2,max_ (kPa)	79.2 (50.7, 103.3)	150.4 (90.2, 192.7)	<0.001	0.793	130.4	65.4	93.9
	Es_2,mean_ (kPa)	21.2 (10.7, 28.8)	41.5 (35.2, 54.6)	0.008	0.715	39.7	66.7	78.6
	Es_2,min_ (kPa)	4.2 (2.4, 6.9)	5.3 (2.4, 8.3)	0.537	0.538	4.7	55.9	51.8
	Es_2,sd_ (kPa)	13.8 (8.7, 16.4)	25.3 (12.4, 33.2)	0.012	0.763	23.9	54.2	91.6
	Els_2,max_ (kPa)	76.2 (48.2, 109.5)	157.3 (93.9, 206.2)	0.015	0.817	116.3	65.8	93.2
	Els_2,mean_ (kPa)	23.2 (13.5, 30.5)	35.8 (29.3, 47.4)	0.017	0.717	42.6	65.3	74.4
	Els_2,min_ (kPa)	4.7 (3.0, 7.1)	6.2 (4.3, 8.6)	0.060	0.594	5.1	63.6	55.7
	Els_2,sd_ (kPa)	16.4 (9.8, 18.4)	26.3 (16.8, 33.1)	0.013	0.770	24.6	56.8	91.4
Shell 3mm	Es_3,max_ (kPa)	79.2 (50.7, 103.3)	162.7 (85.5, 199.4)	<0.001	0.750	127.8	68.5	90.1
	Es_3,mean_ (kPa)	20.4 (9.5, 26.3)	40.8 (33.1, 57.9)	0.018	0.684	36.4	64.2	71.5
	Es_3,min_ (kPa)	3.9 (2.2, 6.8)	4.8 (2.5, 7.9)	0.627	0.542	4.5	59.3	54.2
	Es_3,sd_ (kPa)	14.1 (8.6, 17.2)	22.7 (12.5, 31.4)	0.010	0.759	23.6	58.2	90.2
	Els_3,max_ (kPa)	75.7 (48.5, 112.4)	157.3 (95.3, 212.3)	0.018	0.814	115.2	65.1	87.4
	Els_3,mean_ (kPa)	23.5 (14.0, 31.8)	35.2 (28.4, 48.3)	0.022	0.674	41.3	65.3	74.4
	Els_3,min_ (kPa)	4.3 (2.8, 6.6)	6.2 (4.3, 8.6)	0.053	0.602	4.7	64.2	56.8
	Els_3,sd_ (kPa)	16.8 (10.2, 19.1)	27.9 (17.3, 35.7)	0.011	0.783	25.4	56.4	92.4
BI-RADS combined with the stiff rim sign		-	-	-	0.946	>4a with stiff rim sign	92.31	90.91

BI-RADS: breast imaging reporting and data system, Emax: maximum Young's modulus value, Emean: mean Young's modulus value, Emin: minimum Young's modulus value, Esd: standard deviation of Young's modulus value. The maximum, mean, minimum Young's modulus (YM), and the YM standard deviation in lesion, shell, and the region containing lesion and shell were marked as E_max_, E_mean_, E_min_, and E_sd_ for lesion; Es_max_, Es_mean_, Es_min_, and Es_sd_ for shell; and Els_max_, Els_mean,_ Els_min_, and Els_sd_ for lesion plus shell, respectively. The Es and Els of different shell thicknesses were distinguished by *n* (*n* = 1,2,3), namely Es*_n_* and Els*_n_* (eg, Es_1,max_ and Els_1,max_ for 1mm shell).

**Table 3:** BI-RADS classification based on ultrasound or ultrasound combined with the stiff rim sign

BI-RADS classification	Ultrasound		Ultrasound combined with the stiff rim sign	
	Benign (n=132)	Malignant (n=78)	Benign (n=132)	Malignant (n=78)
3	56	0	107	0
4a	59	17	17	6
4b	17	15	8	22
4c	0	31	0	9
5	0	15	0	41

BI-RADS: breast imaging reporting and data system.

**Table 4:** Coincidence rates of pathological T stage evaluated by SWE and conventional ultrasound

pT stage	Number	Ultrasound				Coincidence rate (%)	SWE				Coincidence rate (%)	*P* value
		T1	T2	T3	T4		T1	T2	T3	T4		
T1	20	14	6	0	0	70.0%	17	3	0	0	85.0%	0.256^*^
T2	49	20	29	0	0	59.2%	3	45	1	0	91.8%	<0.001^#^
T3	9	0	6	3	0	33.3%	0	3	6	0	66.7%	0.157^*^
T4	0	0	0	0	0	-	0	0	0	0	-	
Total	78	34	41	3	0	58.9%	20	51	7	0	87.2%	<0.001^#^

* for Fisher's exact test, ^#^ for Chi-squared test. SWE: shear wave elastography, pT: pathological T stage.

**Table 5:** PPVs of pathological T stage evaluated by SWE and conventional ultrasound

PPV	T1	T2	T3	T4
Ultrasound	41.2% (14/34)	70.7% (29/41)	100% (3/3)	-
SWE	85.0% (17/20)	88.2% (45/51)	85.7% (6/7)	-
*P* value	0.002^#^	0.035^#^	0.490^*^	

* for Fisher's exact test, ^#^ for Chi-squared test. SWE: shear wave elastography, PPV: positive predictive value.
